# Unmasking the hidden impact of viruses on tuberculosis risk

**DOI:** 10.1016/j.it.2024.07.008

**Published:** 2024-08-23

**Authors:** Fatoumatta Darboe, Josephine F. Reijneveld, David P. Maison, Leonardo Martinez, Sara Suliman

**Affiliations:** 1 Zuckerberg San Francisco General Hospital, Division of Experimental Medicine, University of California San Francisco, San Francisco, CA, USA; 2 Boston University School of Public Health, Department of Epidemiology, Boston, MA, USA; 3 Chan Zuckerberg Biohub, San Francisco, CA, USA; 4 These authors contributed equally; 5 These authors contributed equally

## Abstract

Tuberculosis (TB) is a leading cause of mortality from an infectious disease. In this opinion article, we focus on accumulating scientific evidence indicating that viral infections may contribute to TB progression, possibly allowing novel preventive interventions. Viruses can remodel the mammalian immune system, potentially modulating the risk of reactivating latent microbes such as *Mycobacterium tuberculosis* (*Mtb*). Evidence is mixed regarding the impact of emergent viruses such as SARS-CoV-2 on the risk of TB. Therefore, we posit that important knowledge gaps include elucidating which viral families increase TB risk and whether these provide unique or shared immune mechanisms. We also propose potential future research to define the contribution of viruses to TB pathogenesis.

## Evidence supporting the contribution of viral infections on TB progression

Globally, TB is a leading cause of human mortality from an infectious disease caused by a single pathogen, ***Mycobacterium tuberculosis* (*Mtb*)** (see Glossary) [1]. However, only an estimated 5–10% of individuals exposed to *Mtb* develop TB [2]. Many factors are known to increase the risk of progression to TB, such as recent *Mtb* infection [3–5], sex [6], and comorbidities [7,8]. Several reports suggest that viral infections increase the risk of progression from *Mtb* infection to disease [9,10]. Innate antiviral responses, such as elevated type **I interferon (IFN)** signaling, are associated with TB risk and bacterial load following *Mtb* infection [11–18]. Blood transcriptional signatures of TB disease [13], or prospective risk of TB progression [14], show elevated type I IFN responses compared with asymptomatic controls. In C57/BL6 mice, pre-infection of mice with influenza A virus (IAV) increased *Mtb* bacterial load and mortality [12]. However, this effect was obliterated in IFNα/β receptor knockout (*Ifnar*^−/−^) mice [12]. The most well-known virus to increase TB risk is human immunodeficiency virus (HIV), where HIV-1-mediated CD4^+^ T cell depletion compromises functional T cell responses against *Mtb* antigens [19–21]. Although HIV-1 provides a dramatic example of a chronic viral infection that increases TB risk and mortality, we argue that this interaction between viruses and TB risk is not unique to HIV-1.

Emerging evidence supports a general role for viral infections in increasing TB risk in humans. **Cytomegalovirus (CMV)**, another chronic virus, has also been associated with elevated TB risk [22]. In addition, TB incidence was reported to be higher in persons with untreated hepatitis C virus (HCV) infection compared with untreated individuals [23]. Pre-infection of mice with lymphocytic choriomeningitis virus (LCMV) clone 13 (Cl13) 21 days before *Mtb* infection increased *Mtb* bacterial load in the lung and spleen, and increased overall mortality compared with LCMV-naive mice [24,25]. Some patients with acute virus infection, such as severe acute respiratory syndrome coronavirus 2 (SARS-CoV-2), can develop unexplained long-term sequelae such as neurocognitive and gastrointestinal symptoms [26]. In a small cohort of patients with post-acute sequelae of coronavirus disease 2019 (COVID-19), symptomatic persistence was associated with residual tissue-specific SARS-CoV-2 viral reservoirs detected in biopsied gut epithelial tissues, as evidenced *in situ* hybridization of single-stranded SARS-CoV-2 RNA, indicating active viral replication [27]. Subsequently, inflammation caused by acute viral infections (i.e., ones resolve following infection), such as IAV, may transiently increase TB risk, although this has not been systematically studied. Supporting this idea, RISK11, a blood-based 11-gene transcriptional signature predictive of TB risk, was derived from prospective cohorts of *Mtb*-infected persons using RNA sequencing (RNA-seq) analysis; the signature was enriched in individuals infected with upper respiratory tract infections, including IAV, common coronaviruses, and rhinoviruses [28]. Seasonal fluctuations in TB risk have been attributed to *Mtb* transmission patterns, but may also be caused seasonal viruses [29–34]. Defining which viral families and mechanisms of pathogenesis are associated with higher TB risk, and the durability of increased risk, remain important knowledge gaps. We hypothesize that viruses capable of establishing long-term reservoirs, or triggering sustained local or systemic inflammation, will increase the risk of TB pathogenesis. In this opinion piece we review available evidence of the association between several viruses – including HIV-1, CMV, IAV, SARS-CoV-2, flaviviruses, and measles virus – and TB risk. We also review possible IFN-dependent and -independent mechanisms by which viruses might increase TB pathogenesis. Finally, we outline current outstanding questions for future research.

## HIV-1

HIV-1 is likely the single greatest risk factor for TB progression from an infectious agent; indeed, persons living with HIV-1 (PLWH) are up to 16 times at higher risk of TB progression than people without HIV-1 [8,35–40]. Clinical features of TB presentation depend on the stage of HIV-1 infection and immunodeficiency, captured clinically by CD4^+^ T cell counts [41]. The impact of HIV-1 on TB risk is likely underestimated due to the paucibacillary TB presentation in PLWH, leading to *Mtb* smear and culture negativity [42], and unreliable X-ray presentations [43], particularly with advanced immunosuppression [44].

**Figure 1.**
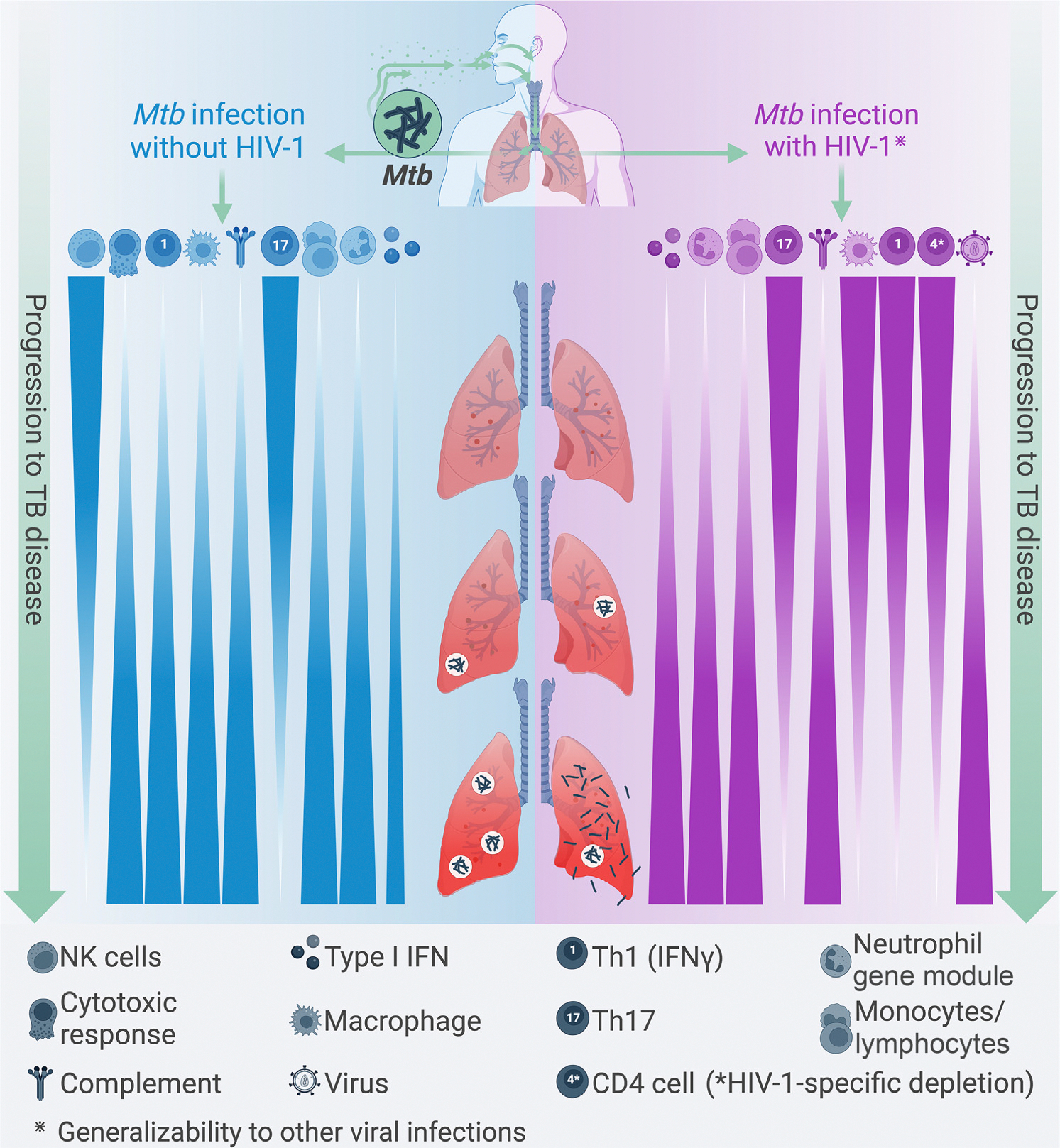
Model of tuberculosis (TB) progression during *Mycobacterium tuberculosis* (*Mtb*) and viral co-infection. The schematic shows TB progression from asymptomatic *Mtb* infection (top) to full TB disease (bottom) in the absence of a viral co-infection (left, blue), or in the presence of a viral co-infection (right, purple). Mechanisms are extrapolated from known immune changes during human deficiency virus 1 (HIV-1) infection, but we hypothesize that they may be generalizable to many chronic viral infections capable of establishing long-term reservoirs. The lungs show high pulmonary cavitation (gas-filled space) in the absence of viral infection, and higher miliary disease (spread via the bloodstream) and dissemination when the virus is present. Immune features associated with TB progression and direction of change are depicted across the TB spectrum. CD4^+^ T cell depletion is specific to HIV-1, but other mechanisms may be generalizable to certain chronic viral infections. See main text for references. Figure adapted from ‘How tuberculosis spreads’ by BioRender.com.

**Figure 2.**
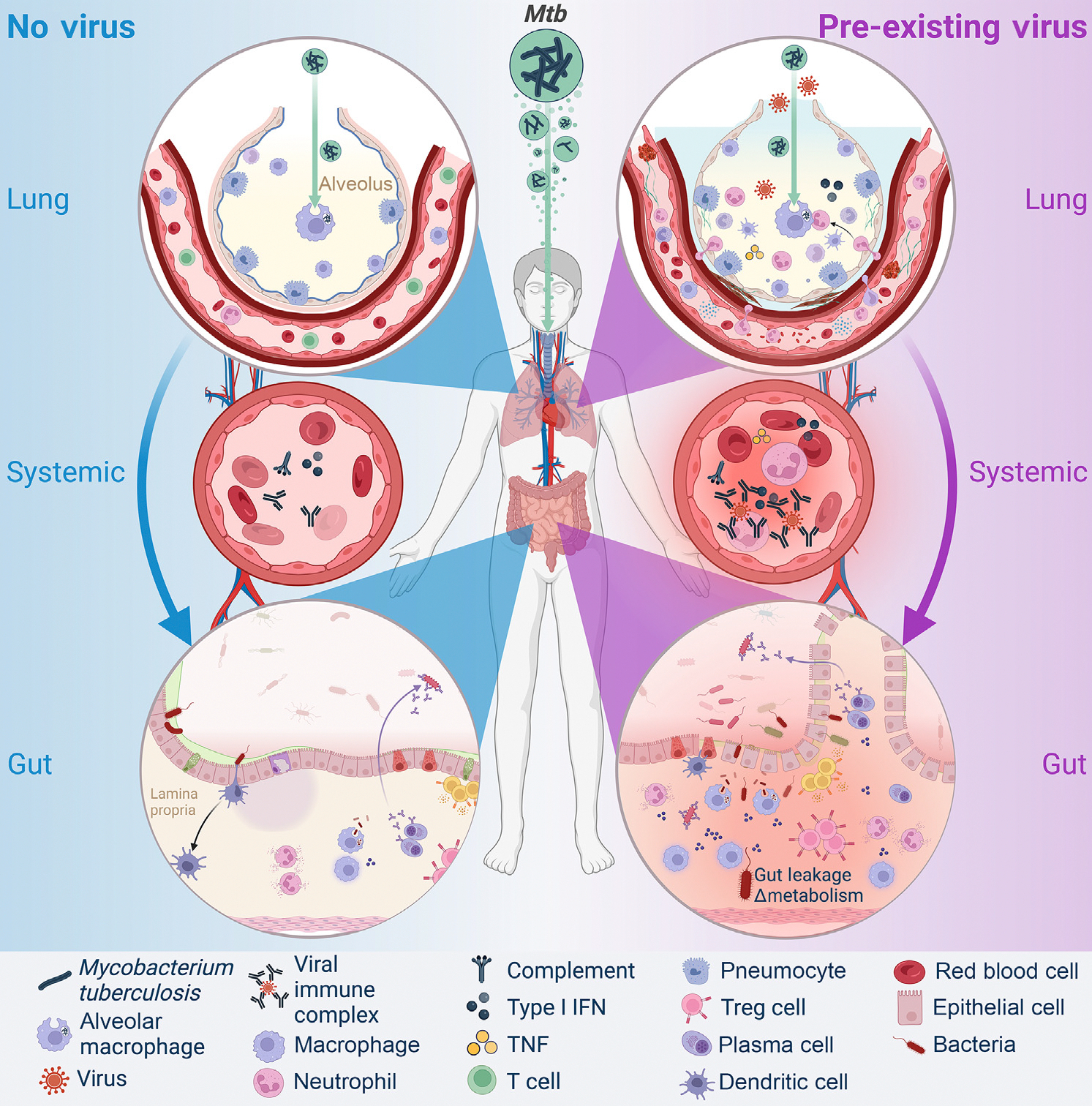
Key figure: Examples of the immunological consequences of *Mycobacterium tuberculosis (Mtb*) co-infection with a pre-existing virus The schematic depicts a scenario of *Mtb* infection of lung alveoli in the presence and absence of a pre-existing viral infection, with systemic and gut consequences. The figure demonstrates aerosol infection with *Mtb* in lung alveoli in otherwise healthy alveoli (left, blue), and alveoli with an established viral infection (right, purple). The left side of the *Mtb* infection demonstrates the systemic effects in circulation, with increasing antibody titers, complement, and type I interferon (IFN) production, resulting in limited to no impact on gut permeability. The right side demonstrates a scenario where *Mtb* enters an alveolus with an established and ongoing respiratory viral infection, illustrating acute respiratory distress syndrome (ARDS) features such as fluid and fibrosis in the interstitial space, immune infiltration into the alveolus, and coagulation in the capillaries. The right-side infection proceeds with amplified systemic effects and inflammation, with more type I IFN and complement production, increased numbers of immune cells, viral immune complexes, and tumor necrosis factor (TNF) production, resulting in gut leakage and changes in gut bacteria metabolism. See main text for references. Figure created with Biorender.

HIV-1 infection increases TB risk through several mechanisms, including immune hyperactivation, exhaustion of the immune system, systemic inflammation, and depletion of CD4^+^ T cells, leading to a loss of TB control [45]. *Mtb* co-infection is also associated with increased viral replication [46], where HIV-1 replicates more in HIV-1/*Mtb* co-infected macrophages than in neutrophils, instigating a positive feedback loop that increases both *Mtb* and HIV-1 virulence, as evidenced from confocal microscopy imaging [47]. Type I IFNs are linked to acute/early HIV-1 infection [48] and HIV-1 progression [49]; they can also drive *Mtb*-induced cell death [18] and TB pathogenesis [16,50]. Moreover, in non-human primates (NHPs) infected with simian immunodeficiency virus (SIV), blocking the IFNα/β receptor (IFNAR) via an IFN-I receptor antagonist increased blood SIV reservoir size, decreased the number of proliferating Ki67^+^ CD4^+^ T cells, and accelerated CD4^+^ T cell depletion and progression to acquired immunodeficiency syndrome (AIDS). This suggested that type I IFN had a protective effect against disease progression [51]. In humans, HIV-1 viremia has been documented to be inversely associated with IFN production in blood, suggesting that type I IFN is linked to viral control [52]. Infection of human monocyte-derived DCs with *Mtb* induced the release of IFNβ followed by IFNα in culture supernatants (detected via enzyme-linked immunosorbent assay, ELISA) [53], raising the possibility that *Mtb*-mediated type I IFN induction might protect against HIV-1 replication. However, increased HIV-1 replication in the lungs of *Mtb/*HIV-1 co-infected persons compared with controls – likely due to recruitment of *Mtb*-specific activated CD4^+^ T cells to the lung – argues against this hypothesis [54]. Moreover, HIV-1 infection depletes *Mtb*-specific **Th1/Th17** cells [21,55] and **Th22** CD4^+^ T cells in human blood and in bronchoalveolar lavage samples [20]. This suggests that HIV-1 infection can cause selective depletion of *Mtb*-specific CD4^+^ T cell responses that are thought to be protective against TB pathogenesis [56]. Overall, HIV-1 infection may increase the risk of TB through several mechanisms, independently of immunodeficiency status or selective depletion of activated CD4^+^ T cells. In fact, despite early initiation of antiretroviral therapy, viral suppression, and normalization of CD4^+^ T cell counts in PLWH, these individuals were reported to retain a higher risk of developing TB than the general population [38]. We propose that mechanisms influencing some of these outcomes might be shared, to some extent, with other chronic viral infections that do not dramatically impart immunodeficiency (Figure 1). This is because viral infections elicit several tissue-specific and systemic inflammatory effects that could increase *Mtb*-mediated pathogenesis (Figure 2, Key figure). For instance, LCMV (Clone 13) infection in mice establishes chronicity via the differential kinetics of the host immune response to the virus, causing elevated type I IFN in the lung and delayed T cell priming, leading to poor control of *Mtb* [24]. In the following, we review some of the inflammatory sequelae induced by other common viruses that may increase susceptibility to TB.

## CMV

Among common chronic viral infections, CMV, a ubiquitous double-stranded DNA herpesvirus, has been suggested to impact clinical outcomes of *Mtb* infection [57,58]. Epidemiological human data show conflicting results on the association between CMV and *Mtb* infections, with some studies showing positive associations [59] while others show null results [22]. For TB progression risk, CMV acquisition was suggested to increase the risk of progression to disease in human cohorts ranging in size from 250 to 2174 participants [22,58,60–63]. In a prospective South African birth cohort, there was a significant positive association between CMV infection in the first year of life and elevated TB risk in childhood (adjusted hazard ratio of 3.2) [22]. Of note, CMV-exposed individuals in TB-endemic settings have higher human leukocyte antigen (HLA)-DR expression on *Mtb*-specific CD4^+^ T cells than in non-TB-endemic settings, consistent with CMV-induced aberrant T cell activation, and association with risk of TB disease [64]. In a cohort of South African bacille Calmette–Guérin (BCG)-vaccinated infants, frequencies of CMV-specific HLA-DR^+^ CD4^+^ T cells detected using enzyme-linked immunosorbent spot (ELISpot) assays (indicating prior exposure to CMV) were not associated with TB risk [64]. By contrast, CMV-specific T cell responses were associated with higher frequencies of HLA-DR^+^ CD8^+^ T cells [64], and the presence of these activated, IFNγ-secreting T cells, was linked to TB risk [64,65]. A follow-up study in the same cohort (49 TB cases and 129 matched healthy controls), showed that infants with a positive CMV-ELISpot IFNγ response had a moderately higher TB risk (odds ratio: 2.2), and shorter time to TB diagnosis compared with their CMV-negative counterparts [63]. However, IFNγ responses against Epstein–Barr virus (EBV), another herpesvirus, were not associated with the risk of TB [63]. These studies did not distinguish between congenital and postnatal CMV infections, which could impart different immunological effects, such as the specific expansion of Vγ9^+^ fetal γδ T cells following congenital CMV infection *in utero*, as previously reported [66]. Thus, studies show that the two types of CMV infection may lead to different outcomes following *Mtb* infection. Collectively, these studies suggest a moderate association between CMV infection and TB risk. However, ruling out shared epidemiological risk factors will require careful study designs to assess causality. It will also be important to rule out the impact of non-biological confounders such as low socioeconomic status. Furthermore, it is important to understand the unique impact of other common herpesviruses, including EBV and herpes simplex virus (HSV), on TB pathogenesis.

Despite epidemiological evidence supporting a moderate impact of CMV on TB risk, there are no preclinical models to study this association or its underlying biological mechanisms in the absence of confounding epidemiological factors [57]. In CMV-discordant monozygotic twins, CMV explains most non-heritable heterogeneity in immune phenotypes [67], opening many hypotheses for how CMV infection could elevate TB risk. CMV also expands specific antigen-specific memory natural killer (NK) cell subsets, also known as adaptive NK cells, characterized by their expression of Ly49H in mice [68], and CD94-NK cell group 2 isoform C (NKG2C) in humans [69], with unknown relevance to *Mtb* control. It is unclear how these CMV-expanded NK subsets could modulate TB risk. In a meta-analysis of human cohort studies, higher numbers of blood NK cells were associated with protection from TB in *Mtb*-exposed individuals [70]. It is unknown, however, whether CMV-induced adaptive NK cell subsets modulate the risk of TB progression [71,72]. One hypothesis is that the reported increased CMV-mediated risk of TB could be partly mediated by the expansion of NK cell clusters that do not control *Mtb*, although this remains conjectural and warrants further investigation. Another interesting finding is that human CMV expresses its own interleukin (IL)-10 homolog, encoded by open reading frame (ORF) UL111a [73]. CMV-encoded viral IL-10 was reported to drive immunosuppression by polarizing primary human CD14^+^ monocytes into a CD163^+^ anti-inflammatory M2-like macrophage phenotype [74]. Evidence from lung transplant human recipients suggests that CMV can replicate in the lung [75], thus raising the possibility that CMV might contribute to an immunoregulatory environment which could be associated with a local loss of *Mtb* control, as visualized in the granulomas of patients with TB by using multiplexed ion beam imaging by time of flight (MIBI-TOF) [76]. Although monocytes are not productive for CMV viral replication, CMV induces monocyte differentiation into macrophages, which are permissive for CMV viral replication [77], raising the possibility that CMV-infected macrophages might be susceptible to *Mtb* infection. Nonetheless, these possibilities remain speculative. Accordingly, there is an unmet need to develop suitable and robust preclinical models of co-infection with *Mtb* and CMV or other herpesviruses to define whether and how they impact the risk of TB [57].

## IAV

Respiratory viruses can impact both the local response to *Mtb* in the shared lung environment, or systemically, through proinflammatory mediators. Early evidence from 1947 showed that concomitant infection with *Mtb* and either the pneumonia virus of mice (PVM) or mouse-adapted IAV strain (**PR8**) accelerated the formation of tuberculous lung lesions in *Mtb*-infected mice [78]. Later experiments showed that pre-infection of C57BL/6 mice with IAV PR8 strain 28 days before *Mtb* increased both lung *Mtb* loads and mortality by day 120 post-*Mtb* infection [12]. It is noteworthy that infection with two IAV subtypes (Cal/09 and X31) on days 1 and 14 after *Mtb* infection, respectively, led to higher *Mtb* bacterial loads than controls. Importantly, IFNαβR^−/−^ (*Ifnar1*^−/−^) mice that were infected with *Mtb* alone or co-infected with *Mtb* and the two IAV strains exhibited comparable mycobacterial loads, suggesting that the detrimental impact of *Mtb*/IAV co-infection was mediated via type I IFN signaling [12]. Another study showed that the effects of *Mtb*/IAV co-infection on *Mtb* control in mice (e.g., increased bacterial loads, reduced survival, impaired *Mtb*-specific CD4^+^ T cell responses, and pulmonary macrophage accumulation with increased arginase-1 production) were rescued upon IL-10 neutralization [79]; this was explained in part by prior reports indicating that type I IFN induced IL-10 expression during *Mtb* infection [80,81]. Collectively, these studies suggest that IAV infection can create a lung environment that may not be compatible with *Mtb* control.

In a Gambian cohort of 282 TB patients, participants co-infected with *Mtb* (defined by the **GeneXpert molecular test** and/or mycobacterial culture) and IAV (assessed by RT–PCR) showed a higher mortality risk than patients with pulmonary TB alone [82]. The presence of IAV RNA in the sputum of TB patients was associated with a higher *Mtb* bacterial load, suggesting that an IAV co-infection in TB patients compromises their ability to control *Mtb* [82]. Moreover, a study of 220 483 latently *Mtb*-infected participants in Korea showed that TB incidence was higher in IAV-infected individuals with untreated latent *Mtb* infection compared with IAV-uninfected counterparts [83]. However, in a retrospective study in Indonesia, no differences in the seroprevalence of A/H3N2 IAV (indicating recent IAV infection) were observed between TB patients (*n* = 111) and asymptomatic latently *Mtb*-infected controls (*n* = 111) [9]. In South Africa, reports of patients diagnosed with both TB and IAV (*n* = 42) showed a greater risk of mortality than those diagnosed with either TB (*n* = 1092) or IAV (*n* = 239) alone [84]. Influenza disease was also associated with excess mortality among pulmonary TB patients with and without HIV-1 infection, compared with IAV-infected controls [85]. Therefore, evidence from animal models and human cohorts supports the hypothesis that IAV infection can increase TB incidence and mortality. The looming threat of zoonotic and possible human-to-human transmission of H5N1 IAV [86], with a 50% case fatality rate, raises the concern that *Mtb*-infected individuals could be especially susceptible to TB progression, and present with high disease severity and mortality. Therefore, robust experimental models are urgently needed to elucidate the immunological interactions between IAV and *Mtb*, and the effects on the host immune system.

## SARS-CoV-2

Like IAV, SARS-CoV-2 shares a similar transmission route with *Mtb*, thus increasing the likelihood of modulating antimycobacterial immunity and susceptibility to *Mtb* infection in the lung (Table 1). A recent study showed that both alveolar and activated interstitial macrophages were highly susceptible to SARS-CoV-2 infection in human lung slices *in vitro* [87]. Activated interstitial macrophages showed significantly higher viral replication and induction of IFN-stimulated genes (ISGs) than alveolar macrophages, which showed lower replication and inflammation, as evidenced by multiplexed single-molecule fluorescence *in situ* hybridization (smFISH) of SARS-CoV-2-infected lung slices detecting host RNA, and SARS-CoV-2 positive and negative RNA strands [87]. Another recent study showed that *Mtb* infects alveolar macrophages first, followed gradually by recruited macrophages, which become the predominant site of *Mtb* infection in C57BL/6 mice infected via the aerosol route [88]. In line with this, SARS-CoV-2 infection enhanced the recruitment and activation of interstitial macrophages to the lungs in rhesus macaques [89]. Therefore, it is reasonable to speculate that SARS-CoV-2-activated interstitial macrophages might be directly susceptible to *Mtb* infection; they might also increase local type I IFN signals associated with higher *Mtb* pathogenesis, as evidenced from the transcriptional analysis of ISGs in myeloid cells recruited to the lungs [16,17,50]. In the following, we review the existing evidence for the known immunological interactions between SARS-CoV-2 and *Mtb*.

Several studies tested the impact of SARS-CoV-2/*Mtb* co-infection *in vivo* (Table 1). One of the main mouse models used to study SARS-CoV-2 is K18-hACE2 transgenic mice which express the human SARS-CoV binding receptor (human angiotensin-converting enzyme 2, hACE2), rendering these animals highly susceptible to lethal infection [90]. Mice pre-infected with SARS-CoV-2 showed no differences in mortality following *Mtb* infection compared with SARS-CoV-2-uninfected mice [91]. Counterintuitively, several studies showed that prior *Mtb* infection partially protected humanized K18-hACE2 mice against SARS-CoV-2 infection by significantly reducing viral loads in the lungs [91–93]. These observations suggested that *Mtb* pre-infection might create a protective milieu against SARS-CoV-2; however, the evidence supporting this hypothesis beyond SARS-CoV-2/*Mtb* co-infection studies in mice is limited. Another study demonstrated that mice pre-infected with a sublethal dose of SARS-CoV-2 before or after aerosol *Mtb* exposure did not exacerbate *Mtb*-associated lung pathology or bacterial loads [93]. The magnitude of pre-existing *Mtb* restriction on early SARS-CoV2 replication correlated with *Mtb* dose; also, it was not dependent on Toll-like receptors (TLR) 2 or TLR9, or on IFN-I signaling, because the *Mtb*-driven sustained protective effect in *Ifnar*^−/−^, *Tlr2*^−/−^, *and Tlr9*^−/−^ mice was also observed in wildtype C57BL/6 mice [93]. Conversely, another study showed that SARS-CoV-2 moderately increased *Mtb* colony forming units (CFUs) in the spleen and liver, but not in the lungs when K18-hACE2 mice were pre-infected with *Mtb* followed by SARS-CoV-2 after 4–8 weeks [91]. These findings seem to suggest that SARS-CoV-2 infection might modulate the lung microenvironment to make it less restrictive for *Mtb* infection, allowing *Mtb* to replicate and disseminate to extrapulmonary organs [91]; however, there is heterogeneity in results across studies, which may be due to the order in which a specific pathogen or other infects the host first. Conversely, some of these mouse studies suggest that pre-infection with SARS-CoV-2 does not increase *Mtb* susceptibility; therefore, this observation does not support our proposed hypothesis that SARS-CoV-2 infection enhances susceptibility to *Mtb* infection. Nevertheless, in a study of hospitalized patients from South Africa, latently *Mtb*-infected patients diagnosed with COVID-19 exhibited lower blood frequencies of *Mtb*-specific CD4^+^ T cells (defined by the expression of IFNγ, tumor necrosis factor (TNF), or both, after stimulation with *Mtb* peptides), than historic pre-pandemic patients [94]. This suggested the possibility that COVID-19 might reduce the magnitude of *Mtb* antigen-specific T cell responses and increase subsequent susceptibility to TB disease, although this question has not been systematically studied in controlled epidemiological studies. Several factors may help explain the reported discrepancies in these studies, including the presence of other co-infections such as CMV or HIV-1, the timeline of *Mtb* and SARS-CoV-2 infections, the animal and *in vitro* models used, as well as the selected *Mtb* and SARS-CoV-2 inoculum doses [87,90–94]. Consequently, more research is required to establish whether SARS-CoV-2 can disrupt *Mtb*-infection outcomes, such as the presence of *Mtb*-containing granulomas leading to reactivation and dissemination of *Mtb*, as well as the worsening of pathology during TB [95].

## Emerging and re-emerging viral infections with unknown impact on TB risk

### Flaviviruses

Flaviviruses are single-stranded RNA viruses, which are arboviruses (i.e., arthropod-borne viruses). It is of concern that the incidence of dengue fever, caused by the dengue virus (DENV) serotypes 1–4, has been steadily rising globally [96]. Whereas dengue fever used to be largely restricted to jungles and rural areas, the incidence is slowly increasing in urban areas and new regions, including the USA, due to increased travel and climate change [96]. This rise in DENV will lead to novel immunological interactions in people exposed to both DENV and *Mtb* which remain unexplored. Several immunological interactions may be at play. For instance, the Fc gamma receptors FcγRI and FcγRII were reported to enhance cellular uptake of DENV infectious immune complexes through mutagenesis of immunoreceptor tyrosine-based activation motifs (ITAM) in COS-7 fibroblast cell line receptors [97]. TB progression is associated with higher expression of FcγRI and FcγRII on monocytes [14], raising the possibility that these receptors could ligate viral antigen–antibody immune complexes [98]. We predict that this ligation would increase antibody-dependent enhancement where DENV immune complexes could increase viral entry into cells, leading to worse symptoms and inflammation [97]. This might in turn increase systemic type I IFN responses, likely associated with TB risk [13]. Other flaviviruses endemic to many parts of the world include Chikungunya, Zika, West Nile, Yellow Fever, and Ebola [99]. Recent transmission of all these viruses has been documented in TB-endemic settings, raising questions about the effects of *Mtb* co-infection with flaviviruses on the human immune system. The extent of flavivirus transmission is likely underestimated. Studies estimating current and persistent infections with flaviviruses using **viral metagenomics**, or history of exposure to flaviviruses using serological tests, can help uncover the epidemiological associations between flavivirus transmission and TB risk.

### Measles virus (MV)

MV causes a vaccine-preventable disease; it is a highly contagious airborne virus with a high case fatality rate [100] and rising incidence [101]. MV infection causes ‘immune amnesia’ where MV infects both B cells and memory T cells, rapidly depleting them and replacing them with MV-specific cells [102]. This immunosuppression increases the risk of secondary opportunistic infections. There is little epidemiological evidence supporting the hypothesis that MV increases TB risk. A recent study from Korea showed a lower incidence of TB following an MV outbreak in humans and concluded that MV infections might have a mild preventative effect against TB [103]. Hence, it is important to design studies that disentangle common risk factors that predispose individuals to both TB and measles, from the direct impact of co-infection [104,105]. With the unfortunate reemergence of MV, understanding these interactions is urgent.

## Possible mechanisms by which viral infections could increase TB risk

### Type I IFN-dependent mechanisms of TB risk

Inflammation caused by elevated signaling through type I IFN receptors has been associated with TB risk and higher bacterial load after *Mtb* infection, based on data from mouse studies and human cohorts [11–15]. Intranasal administration of polyinosinic:polycytidylic acid (Poly:IC), a type I IFN agonist, was sufficient to increase *Mtb* bacterial loads and exacerbate lung pathology in *Mtb*-infected mice [106]. Older studies using different knockout mouse strains, as well as *Mtb* infection of mouse bone-marrow-derived macrophages, demonstrated induced expression of genes associated with *Mtb* infection, including the gene encoding inducible nitric oxide synthase (iNOS); this occurred independently of TLR2/4 or IFNγ expression, but required expression of IFNAR and STAT1, the transcription factor downstream of type I IFN activation [107]. Activation of type I IFN was also observed in *Mtb*-infected primary human macrophages, limiting IL-1β production [108]. IL-1β is an important cytokine that curbs *Mtb* growth, as evidenced from experiments in mice lacking IL-1α, IL-1β, or IL-1R [109]. Recent work demonstrated that uninfected plasmacytoid dendritic cells (pDCs) and *Mtb*-uninfected interstitial macrophages were dominant sources of IFN in *Sp140*^−/−^ *Mtb*-infected mice [16,110]. These mice have a deletion in the nuclear body protein SP140-like protein (SP140), which causes higher type I IFN responses [110], rendering *Sp140*^−/−^ mice more susceptible to *Mtb* infection and lethality than C57BL/6 mice [110]. TB susceptibility in the *Sp140*^−/−^ mouse model was reversed by IFNα receptor knockout (*Ifnar*^−/−^ mice) [16,50]. These mouse studies indicated that TB pathology was partly mediated via type I IFN signaling. In human cohorts, type I IFN signaling is strongly correlated with upregulated ISGs in patients with TB [13,14], suggesting that type I IFNs are associated with TB disease progression and disease severity [13,14].

### Type I IFN-independent mechanisms of TB risk

**Figure 3.**
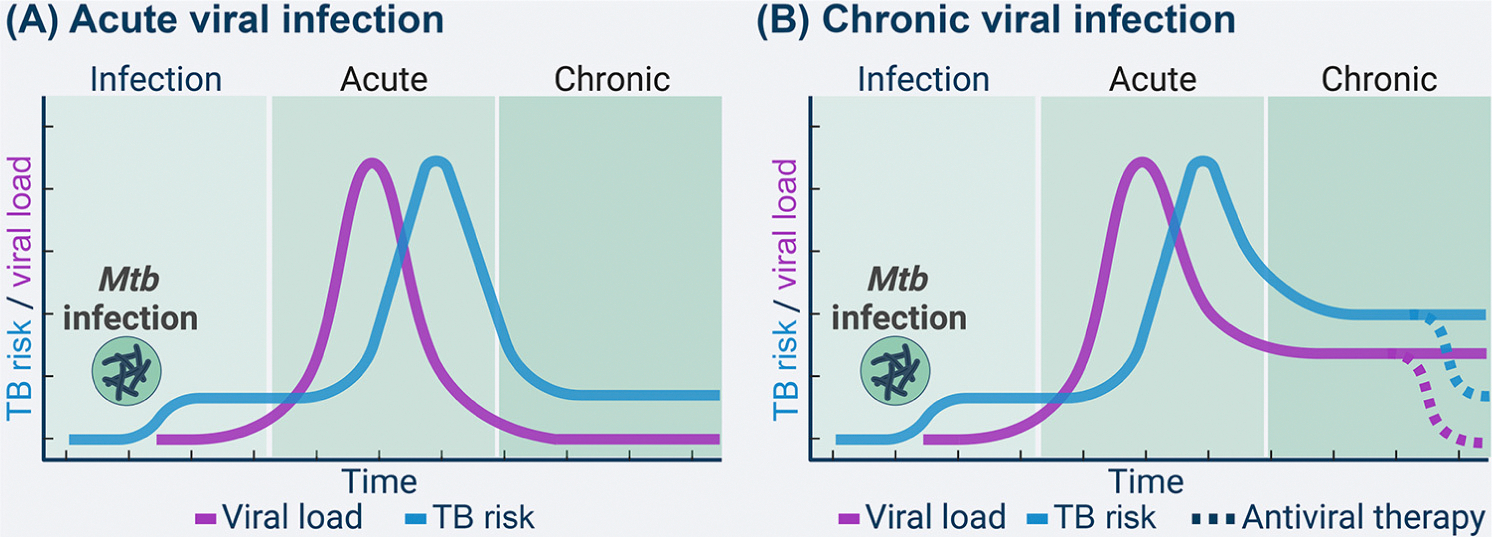
Two-hit model of viral infections and increased tuberculosis (TB) risk. Initial *Mycobacterium tuberculosis (Mtb*) infection constitutes the first hit to increase TB risk in both acute (A) and chronic (B) viral infections. (A) In acute viral infections, where the virus is cleared naturally or therapeutically, we hypothesize that TB risk is only transiently increased in a small window of time, during and after the viral infection. TB risk is likely to subside once the viral load decreases. (B) In chronic viral infections, the virus establishes a persistent long-term reservoir after the acute stage of infection. This may result in a persistently elevated TB risk due to increased inflammation as well as both systemic and tissue-specific type I interferon (IFN) signaling. Antiviral therapies capable of clearing this reservoir may then restore TB risk to previral infection levels (dashed lines). See main text for references. Figure created with Biorender.

Gene signatures that have been characterized in HIV-1-infected persons better predict TB risk in viremic individuals [111], because both cases and controls bear a high viral load. One of the most prominent signatures is the activation of genes associated with the complement pathway [111,112]. Although many signals can activate the complement pathway, one plausible mechanism bridging viral infections and TB risk is the formation of viral immune complexes that can trigger the C1q complement fragment. Enrichment of antiviral modules in blood samples from TB patients relative to those at high risk of progression might be an indication that the viral infection can precipitate TB risk in a two-hit model of pathogenesis (Figure 3) [113,114]. The tubercle bacillus is the necessary or initiating cause, but some level of reduced host resistance, such as virus-induced decreased cellular immunity and increased systemic inflammation, might become main instigating factors. Overall, there are many unexplored mechanisms by which viral infections may contribute to TB disease, warranting additional studies.

## Implications and knowledge gaps

Our hypothesis states that viruses capable of establishing a long-term reservoir, and sustaining a pro-inflammatory milieu, are more likely to drive sustained increased TB risk. Whereas HIV-1, CMV, and IAV have positive associations with TB risk, the evidence remains unclear for other viruses. To derive epidemiological evidence to test the hypothesis that viral infections might increase TB risk, understanding which viruses are circulating in different communities, and the extent of transmission in TB-endemic settings is a first step. However, these studies have often relied on technologies with limited throughput using molecular or serological tests to detect specific viruses. The technologies have advanced to use higher-throughput methods, such as metagenomic sequencing [115], and high-throughput seroprevalence platforms, such as **virome serological profiling** [116]. These technologies are prohibitively expensive for mass deployment in large populations. However, limited pilot studies to define which groups of viruses are likely associated with higher TB risk may then allow for a targeted analysis of these viruses in high-risk populations, followed by mechanistic *in vitro* studies and studies in animal models. Preclinical testing of antiviral regimens – such as acyclovir, ganciclovir, valacyclovir, or valganciclovir – as preventative or adjunct therapies for TB might be novel approaches to decrease TB risk, especially if it can be established that viral co-infections increase TB risk or progression. Evidently, systematic preclinical and clinical evidence will be necessary for developing such approaches.

## Concluding remarks

Although surmised for some time and based on substantial supportive immunological and epidemiological empirical evidence, viral infections are an underappreciated and suboptimally validated/reported contributors to TB risk and/or progression (see Outstanding questions). Systematic analysis of the impact of families of viral infections on TB risk can inform novel public health screening strategies for such risk, including screening current or prior viral exposures with molecular or serological tests. These studies can also motivate the preclinical development of novel candidate therapeutic targets, such as antivirals or immunomodulatory agents, to curb or reverse the impact of viral infections on immune pathways that are relevant for TB control. With emerging and re-emerging viral pandemics, a clinical and immunological framework to study the impact of such viral infections on immunological pathways and TB pathology will be highly relevant to controlling TB epidemics.

## Figures and Tables

**Table 1.** Summary of murine studies in SARS-CoV-2 and *Mtb* co-infection^a^

Model	SARS-CoV-2 viral load	Mtb bacterial load	Refs
K18-hACE2 mice were challenged with *Mtb* followed by SARS-CoV-2 (WA1/2020) after 4 or 8 weeks	↓	↑	[91]
K18-hACE2 mice were challenged with *Mtb* followed by SARS-CoV-2 (WA1/2020) after 30 days	↓	=	[92]
C57BL/6 wild-type mice were challenged with *Mtb* followed by SARS-CoV-2 (MA10) after 30 days	↓	=	
K18-hACE2 mice were challenged with SARS-CoV-2 (WA1/2020) followed by *Mtb* after 28 days	ND	=	[93]
K18-hACE2 mice were challenged with *Mtb* followed by SARS-CoV-2 (WA1/2020) after 170 days	ND	=	
K18-hACE2 mice were challenged with *Mtb* followed by SARS-CoV-2 (WA1/2020) after 30–60 days	↓	ND	
C57BL/6 wild-type mice were challenged with *Mtb* followed by SARS-CoV-2 (B.1.351)after 30 days	↓	ND	

a ↑, increased; ↓, decreased; =, unchanged compared with controls; ND, not determined.

## Highlights

Viral co-infections in *Mycobacterium tuberculosis* (*Mtb*)-exposed individuals are potentially a risk factor for tuberculosis (TB) pathogenesis.

Some chronic viral infections, such as human immunodeficiency virus 1 (HIV-1) and cytomegalovirus (CMV), can increase TB risk. However, it is unclear whether different viruses impart unique or shared pathogenic mechanisms.

Viruses modulate both systemic and local tissue environments in ways that may increase the TB risk.

There is an urgent need for experimental models and approaches to help define the impact of emerging and re-emerging viral epidemics on the risk of TB incidence and mortality in TB-endemic settings.

## Significance

Factors associated with tuberculosis (TB) risk remain poorly defined. Studies indicate that viral infections can heighten the risk of transitioning from latent *Mycobacterium tuberculosis* (*Mtb*) infection to TB. Innate antiviral responses, particularly increased type I interferon signaling, correlate with elevated TB risk and bacterial load following *Mtb* infection. HIV-1 illustrates how a chronic viral infection can amplify TB risk and mortality, but the relationship between viral infections and TB risk may extend beyond HIV-1. Identifying specific viral families and pathogenic mechanisms associated with heightened/persistent TB risk remains a key research area.

## Outstanding questions

To help define the impact of viral infections on TB risk, it is important to identify the following.

- Which viral families are associated with higher risk of TB incidence and pathogenesis?
- Can we deploy new tools such as viral metagenomics and virome serological profiling to decipher viral communities associated with TB risk?
- What is the impact of shared routes of viral and *Mtb* transmission on TB risk?
- Are chronic viruses more likely to increase TB risk than acute viruses?
- What is the impact of residual viral reservoirs on TB risk?
- What is the role of persistent viral antigenemia on TB risk?
- Do infections with viruses forming antigen–antibody immune complexes result in complement activation leading to higher TB-associated tissue pathology than with viruses not forming persistent immune complexes?
- Can either *Mtb* or viruses remodel the immune system via trained immunity, resulting in long-term residual susceptibility to co-infection?
- When assessing virome and microbiome composition, what is the impact of concurrent co-infections and microbial composition on TB risk?
- How do we define the impact of viruses within families known to have opposite effects in other disease contexts on TB risk (e.g., CMV and EBV)?
- In terms of mechanisms of pathogenesis, are the effects of viruses on TB risk mediated by co-infection of the same cells or by a bystander effect due to virus-induced inflammation and remodeling of the local and systemic milieu?
- Can we quantify the synergies between co-infection and environmental remodeling caused by co-infection?

## Glossary

Cytomegalovirus (CMV): a common herpesvirus with manifestations ranging from asymptomatic to severe end-organ dysfunction in immunocompromised patients with congenital CMV disease
GeneXpert molecular test: a rapid PCR test that detects *M. tuberculosis* and simultaneously detects *Mtb* and resistance to the antibiotic rifampicin
Interferons (IFNs): cytokines made and released by host cells in response to the presence of several viruses. Type I IFNs include IFNα and IFNβ, the main function of which is to prevent viral replication in cells
*Mycobacterium tuberculosis* (*Mtb*): pathogenic bacteria transmitted via the aerosol route, causing tuberculosis disease, most often affecting the lungs
PR8: a mouse-adapted influenza virus strain, originally A/Puerto Rico/8, serially passaged from an H1N1 clinical isolate in 1934
Th1/Th17: CD4^+^ T helper cell subsets that play an important role in adaptive immune responses. Th1 cells produce IFNγ, TNF, and IL-2 to support cell-mediated responses. Th17 cells primarily produce IL17 and play a crucial role in inflammatory responses against pathogens
Th22: a CD4^+^ T helper cell subset which primarily produces IL-22; it is thought to be involved in wound healing and mucosal defense
Viral metagenomics: the study of viral genetic sequences isolated from uncultured viruses from environmental and clinical samples
Virome serological profiling: the study of antibodies against viral infections, useful for understanding the history of exposure to viral infections
